# Lack of ST2 aggravates glioma invasiveness, vascular abnormality, and immune suppression

**DOI:** 10.1093/noajnl/vdaf010

**Published:** 2025-01-27

**Authors:** Grzegorz Wicher, Ananya Roy, Alessandra Vaccaro, Kalyani Vemuri, Mohanraj Ramachandran, Tommie Olofsson, Rebeca-Noemi Imbria, Mattias Belting, Gunnar Nilsson, Anna Dimberg, Karin Forsberg-Nilsson

**Affiliations:** Science for Life Laboratory, Uppsala University, Sweden; Department of Immunology, Genetics and Pathology, Rudbeck Laboratory, Uppsala University, Uppsala, Sweden; Science for Life Laboratory, Uppsala University, Sweden; Department of Immunology, Genetics and Pathology, Rudbeck Laboratory, Uppsala University, Uppsala, Sweden; Science for Life Laboratory, Uppsala University, Sweden; Department of Immunology, Genetics and Pathology, Rudbeck Laboratory, Uppsala University, Uppsala, Sweden; Science for Life Laboratory, Uppsala University, Sweden; Department of Immunology, Genetics and Pathology, Rudbeck Laboratory, Uppsala University, Uppsala, Sweden; Science for Life Laboratory, Uppsala University, Sweden; Department of Immunology, Genetics and Pathology, Rudbeck Laboratory, Uppsala University, Uppsala, Sweden; Academic Laboratory, Uppsala University Hospital, Uppsala, Sweden; Science for Life Laboratory, Uppsala University, Sweden; Department of Immunology, Genetics and Pathology, Rudbeck Laboratory, Uppsala University, Uppsala, Sweden; Department of Hematology, Oncology and Radiophysics, Skåne University Hospital, Lund, Sweden; Department of Clinical Sciences, Section of Oncology, Lund University, Lund, Sweden; Department of Immunology, Genetics and Pathology, Rudbeck Laboratory, Uppsala University, Uppsala, Sweden; Department of Medical Sciences, Uppsala University, Uppsala, Sweden; Division of Immunology and Respiratory Medicine, Department of Medicine Solna, and Centre for Molecular Medicine, Karolinska Institutet and Karolinska University Hospital, Stockholm, Sweden; Science for Life Laboratory, Uppsala University, Sweden; Department of Immunology, Genetics and Pathology, Rudbeck Laboratory, Uppsala University, Uppsala, Sweden; Division of Cancer and Stem Cells, University of Nottingham Biodiscovery Institute, Nottingham, UK; Science for Life Laboratory, Uppsala University, Sweden; Department of Immunology, Genetics and Pathology, Rudbeck Laboratory, Uppsala University, Uppsala, Sweden

## Abstract

**Background:**

Glioblastoma (GBM) is the most common primary malignant brain tumor in adults, characterized by aggressive growth and a dismal prognosis. Interleukin-33 (IL-33) and its receptor ST2 have emerged as regulators of glioma growth, but their exact function in tumorigenesis has not been deciphered. Indeed, previous studies on IL-33 in cancer have yielded somewhat opposing results as to whether it is pro- or anti-tumorigenic.

**Methods:**

IL-33 expression was assessed in a GBM tissue microarray and public databases. As in vivo models we used orthotopic xenografts of patient-derived GBM cells, and syngenic models with grafted mouse glioma cells.

**Results:**

We analyzed the role of IL-33 and its receptor ST2 in nonmalignant cells of the glioma microenvironment and found that IL-33 levels are increased in cells surrounding the tumor. Protein complexes of IL-33 and ST2 are mainly found outside of the tumor core. The IL-33-producing cells consist primarily of oligodendrocytes. To determine the function of IL-33 in the tumor microenvironment, we used mice lacking the ST2 receptor. When glioma cells were grafted to ST2-deficient mouse brains, the resulting tumors exhibited a more invasive growth pattern, and are associated with poorer survival, compared to *wild-type* mice. Tumors in ST2-deficient hosts are more invasive, with increased expression of extracellular matrix remodeling enzymes and enhanced tumor angiogenesis. Furthermore, the absence of ST2 leads to a more immunosuppressive environment.

**Conclusions:**

Our findings reveal that glia-derived IL-33 and its receptor ST2 participate in modulating tumor invasiveness, tumor vasculature, and immunosuppression in glioma.

**Key Points**

There is widespread IL-33 expression in the mouse glioma microenvironment, primarily in oligodendrocytes. Glia-derived IL-33 and its receptor ST2 participate in modulating tumor invasiveness, tumor vasculature, and immunosuppression in glioma.

**Importance of the Study**

Gliomas share several characteristics with brain injuries, including inflammation, tissue remodeling, and cell migration. The alarmin IL-33, which is released in response to cellular stress or injury, has also garnered attention for its role in glioma progression. However, in other cancer types, IL-33 has been shown to exhibit both pro- and anti-tumorigenic effects. In this study, we investigated whether IL-33 influences the glioma microenvironment and observed widespread IL-33 expression in mouse models of glioma, predominantly in oligodendrocytes. Furthermore, gliomas in mice lacking the IL-33-receptor, ST2, exhibited increased tumor invasion, vascular abnormalization, and immunosuppression. Our findings suggest that glia-derived IL-33 plays a critical role in orchestrating the brain’s anti-tumor response in an ST2-dependent manner. Our study provides new insights into the mechanisms driving glioma aggressiveness, highlighting the interplay between tumor invasiveness, aberrant neovascularization, and the recruitment of immune cells that contribute to an immunosuppressive tumor microenvironment.

Glioblastoma (GBM) is the most prevalent form of malignant primary brain cancer in adults.^1–4^ Despite surgical resection, irradiation, and chemotherapy, the median survival for GBM patients remains a mere 15 months. The aggressiveness of GBM is attributed to invasiveness, aberrant neovascularization, and the recruitment and accumulation of immune cells that foster an immunosuppressive tumor microenvironment.^5,6^ GBM thrives within a complex niche composed of, eg, neurons, astrocytes, oligodendrocytes, and microglia that can become co-opted to promote tumor growth and spread within the brain.^7–9^ Additionally, immune cells such as macrophages and T cells are often suppressed in the tumor microenvironment, further promoting GBM progression. The intermingling between normal brain cells and tumor cells creates a cancer ecosystem, which supports GBM invasion and therapy resistance.^7^

Cytokines are central in mediating the immune response, facilitating cellular communication within the tumor, and regulating the infiltration and activity of immune cells.^10–12^ This shapes the tumor’s growth and its evasion of the immune system. Interleukin-33 (IL-33), a member of the IL-1 cytokine family, has emerged as an important regulator of innate and adaptive immune responses.^13^ IL-33 is primarily localized to the nucleus, and functions as an alarmin, ie, is released during cell stress or injury.^14–16^ IL-33 and its receptor ST2 have been implicated as regulators of glioma growth.^9,17^ However, findings on IL-33’s role in cancer remain complex, with studies presenting conflicting results on whether it functions as a tumor promoter or inhibitor.^18^ Notably, GBM cells with IL-33 that lack its nuclear localization signal are less tumorigenic. These observations suggest that IL-33 may have multiple, context-dependent roles in GBM.^19^

In this study, we examined the role of IL-33 and its receptor ST2 in nonmalignant cells in the glioma microenvironment. Under normal conditions, the expression of IL-33 in the adult central nervous system (CNS) is low.^20–22^ Upon CNS injury or disease, IL-33 derived from glial cells facilitates the release of proinflammatory chemokines from both microglia and astrocytes,^21,22^ leading to recruitment of eg, microglia/macrophages to the injury site. In experimental stroke, IL-33 activation resulted in an improved outcome characterized by reduced glial scarring and a weakened T-cell response.^23^ Tumors exhibit numerous resemblances with tissue damage, such as inflammation, tissue remodeling, and cell migration.^24^ Given similarities between the mechanisms of wound healing and the development of tumors, we asked whether IL-33 participates in shaping the glioma microenvironment. To address this, we explored how the lack of its receptor, ST2, affects glioma aggressiveness.

## Methods

### Analysis Using Public Databases

To compare IL-33 expression levels between normal brain (GSE11882) and GBM, expression data (MAS5.0 - u133p2) from multiple studies (GSE7696, GSE53733, and GSE36245) was compared on the R2 (http://r2.amc.nl) genomics platform.^25–28^ Kaplan–Meier analysis was performed using the same platform from overall survival data available for the TCGA cohort and GSE7696. Briefly, the datasets were divided into 2 groups based on the IL-33 expression. In the order of expression, every increasing expression value is used as a cutoff to create the 2 groups, and the significance was tested using a log-rank test. The most significant expression cutoff for survival analysis was plotted on the Kaplan–Meier curve based on the log-rank test. Statistical analysis was conducted using Prism version 9.5 (GraphPad Software, Inc.).

### Tissue Microarray

Human GBM tissue was obtained with ethical approval (Uppsala 2007/353). The tissue microarray (TMA) used in this study contained 22 GBM tissue cores and 7 tissue cores from nonmalignant brains. Immunohistochemistry was performed using the IL-33 AF3625 antibody 733 (Supplementary Table 1) with an Autostainer 480 instrument (Lab Vision). The staining procedures, slide scanning, and image acquisition were conducted as described.^29^ TMA cores with GBM and control brains labeled with IL-33 were imaged and automated quantification of IL-33 staining intensity was performed using the ScanScope XT system and digital pathology software platform (Aperio Technologies). In the study, the immunohistochemical staining intensity of brain tumor tissue samples was automatically and quantitatively analyzed using the ScanScope XT system. This system categorized the staining intensity of interleukin-33 (IL-33) in both tumor and control tissue samples into 4 distinct groups: negative, weak, intermediate, and strong. The categorization was based on the level of IL-33 expression observed in the tissue samples.^30^

### Mouse Protocol

Six to eight-week-old C57BL/6J mice (*wt*, *n* = 10, Taconic Bioscience), homozygote ST2-deficient mice (*st2*^*-/-*^, *n* = 10, kindly provided by Prof. A. McKenzie, MRC Laboratory of Molecular Biology, Francis Crick Avenue, Cambridge Biomedical Campus, Cambridge CB2 0QH, UK) or immunodeficient nude mice (*Foxn1*^*-/-*^, *n* = 20, Janvier Labs, Le Genest-Saint-Isle, France) of either gender were used as orthotopic syngenic, or xenograft glioma models. All experimental procedures involving animals were approved by the Uppsala Animal Ethical Committee (Ethical permits 5.8.18-14626/2020, C131/15, and C359/12) in accordance with the Swedish Animal Welfare Act.

### Cell Lines

Human GBM-derived cell lines^31^ U3013MG and U3024MG were cultured in Dulbecco’s modified Eagle medium/F12 (10565, DMEM/F12, Gibco, Thermo Fisher) supplemented with B27 and N2 (12587 and 17502, Invitrogen, Thermo Fisher), penicillin-streptomycin (10378, PS, Invitrogen, 1000 U/ml), epidermal growth factor (10 ng/ml, PeproTech, Thermo Fisher), and fibroblast growth factor-2 (FGF-2, 10 ng/ml, PeproTech,) in laminin-coated (L2020, Sigma-Aldrich) 10 cm tissue culture dishes (Corning Primaria). Mouse glioma cell lines GL261-Luc-GFP (gift from Dr. G. Safrany, National Institute of Research, Budapest, Hungary) and CT-2A-Luc-GFP (gift from Dr. T. Seyfried) were cultured in Dulbecco’s modified Eagle medium (21885025, DMEM, Gibco) supplemented with 10% v/v fetal bovine serum (Invitrogen) in poly-d-lysine-coated (A38904, Gibco) 10 cm tissue culture dishes (Corning Primaria). All cells were maintained at 37 °C in a humidified atmosphere containing 5% carbon dioxide. Passage of the cells was performed every 4–5 days when they reached confluence, and the medium was changed accordingly.

### Intracranial Tumor Cell Injections and Tissue Preparation for Staining

Mice were anesthetized and received a stereotaxic injection of 2 µl tumor cells (1 × 10^4^ cells/µl for GL261 and CT-2A or 5 × 10^4^ cells/µl for U3013MG and U3024MG) using a 10 µl Hamilton syringe (Hamilton, Bonaduz, Switzerland). The injection coordinates were 1 mm anterior to bregma, 1.5 mm lateral from the sagittal suture, and at a depth of 2.75 mm. Mice were observed daily for tumor progression-related symptoms, such as kyphotic posture, lethargy, or weight loss >10%. When the score reached the level predetermined by the Ethical Review permit, the mice were sacrificed, and transcardial perfusion was performed with 1X phosphate-buffered saline (PBS, Gibco) followed by 4% paraformaldehyde (PFA, 1-04005). Isolated brains were fixed overnight in 4% formalin solution at 4 °C and cryoprotected in 30% sucrose for 48 hours. The tissue was then sectioned into 10 µm thick coronal sections using a CryoStar NX50 Cryostat (Thermo Fisher). Cryosections with glioblastoma were mounted onto SuperFrost Plus slides (Thermo Fisher) and stored at −20 °C.

### Hematoxylin and Eosin Staining

Cryosections of brain tissue from wild-type (*wt*) and *st2*-knockout mice were stained using Mayer’s hematoxylin (HistoLab, Askim, Sweden) for two minutes, followed by continuous washing under running water for 10 minutes. The sections were then counter-stained with 0.2% eosin (HistoLab) for 10 seconds. Subsequently, the sections were dehydrated with two successive washes in 96% ethanol for 20 seconds and 100% ethanol for 10 minutes. Finally, the sections were treated with xylene (T.J. Baker) for 10 minutes, mounted with Pertex (HistoLab), and air dried.

### Immunofluorescence Staining

Cryosections of brain tissue were thawed and washed in three consecutive cycles of five minutes each with 1X PBS. Subsequently, the sections were incubated in 10X Carbo-Free Blocking Solution (SP-5040, Vector Laboratories) diluted in 1X PBS for 1 hour at room temperature. Primary antibodies (Supplementary Table S1) were diluted 1:200 in the blocking solution, and the samples were incubated overnight at 4 °C. After three consecutive 5-minute washes with 1X PBS, the samples were incubated with secondary antibodies (Supplementary Table 2) diluted 1:200 in blocking solution for 4–5 hours at room temperature. Finally, the samples were washed with 1X PBS and mounted using either ProLong Diamond Antifade Mountant with DAPI (Thermo Scientific) or Fluoromount-G with DAPI (Thermo Scientific).

### Proximity Ligation Assay

Tissue sections from mouse tumor brains were incubated with primary antibodies to ST2 and IL-33, diluted in NaveniFlex (Navinci Diagnostics) primary antibody diluent at 4 ^o^C overnight. The following day, the NaveniFlex proximity ligation assay was conducted using the manufacturer’s instructions (NaveniFlex Tissue GR red, Navinci Diagnostics). To ensure the accuracy of the analysis, control experiments were performed by excluding each primary antibody. The tissue sections were then mounted on glass slides using Fluoromount G and visualized using an epifluorescent microscope at ×40 magnification.

### Mouse Blood Samples and Protein Array

Blood was collected from the heart of anesthetized mice at the experiment endpoint, and allowed to clot at room temperature followed by centrifugation for 15 minutes at 2000 × *g*. The serum was removed immediately, aliquoted, and stored at −20 °C until use. Samples from all mice per genotype per state (control/tumor) were pooled to perform the protein array (ARY028, R&D Systems). The assay was carried out according to the manufacturer’s instructions. The filter layout and the area of the positive control spots were defined and used for all the other spots. Any signal spots falling outside the filter layout were considered artifacts. Quantification of integrated density for the duplicate spots on the filters was done using ImageJ software. Z-scores were calculated for and plotted using Prism version 9.5 (GraphPad Software, Inc.).

### IgG Staining

Tumor sections were stained for endogenous IgG to analyze vessel leakage. Fixed brain sections were washed for 5 minutes with 1X PBS, and staining was performed by incubating the sections with anti-IgG antibodies (Supplementary Table S1) diluted 1:200 for 1 hour at room temperature. Next, the samples were washed with 1X PBS and mounted using Fluoromount-G with DAPI (Thermo Scientific).

### Stereological Quantification

CD31-stained immunofluorescent images were analyzed for blood vessel parameters as described.^32^ Vessel volume, vessel length, mean vessel area, and diameter were determined using a counting frame grid created in Adobe Photoshop 23.0.2.

### Isolation of Immune Cells From Tumor-Bearing Mice

Single-cell suspensions of tumor-bearing mouse brains were obtained by enzymatic dissociation of the brain (excluding the cerebellum). GentleMACS Octo Dissociator (Miltenyi Biotec, Bergisch Gladbach, Germany) along with a tumor dissociation kit (Miltenyi Biotec) were used for the dissociation process as per the manufacturer’s protocol. Myelin depletion was achieved by centrifugation at 2600 rpm (low brake = 2) in a solution of 25% BSA (in PBS) at 4 °C for 20 minutes, followed by the myelin-containing deposit removal. CD45 + immune cells were enriched using Mouse CD45 MicroBeads and Mouse CD8 (TIL) MicroBeads, respectively (Miltenyi Biotec) as per the manufacturer’s protocol. The isolated cells were used for flow cytometry and FACS analysis.

### Flow Cytometry Analysis

CD45 + immune cells isolated from tumor-bearing brains were stained with a fixable viability dye according to the manufacturer’s protocol (BD Biosciences cat #564997). Cells were incubated with anti-mouse CD16/CD32 antibodies (Biolegend, clone 93, #101320) for 15 minutes at 4 °C to block unspecific Fc receptor binding. Next, cells were stained for the surface markers of interest using fluorochrome-conjugated antibodies (Supplementary Table S1) diluted in Brilliant Stain Buffer Plus (BD Biosciences, #566385) for 20 minutes at 4 °C. Nuclear and intracellular proteins were stained using the True-Nuclear™ Transcription Factor Buffer Set (BioLegend, #424401) according to the manufacturer’s instructions. Stained samples were acquired using a CytoFLEX LX (Beckman Coulter). Data were analyzed using FlowJo software version 10.5.3 (FlowJo LLC). The gating strategy for FACS analysis of myeloid and T cells is presented in Supplementary Figures S9 and S10.

### RNA Purification, cDNA Synthesis, and Quantitative PCR

Isolation and purification of RNA from mouse brains with tumors were conducted following the manufacturer’s instructions for the GeneJET RNA Purification Kit (K0731, Thermo Scientific). A modification was made during the cell isolation step, and StemPro Accutase was used instead of trypsin. The RNA concentration was determined using a NanoDrop 2000 spectrophotometer (Thermo Scientific). To synthesize complementary DNA (cDNA), the High-Capacity RNA-to-cDNA Kit (4387406, Thermo Scientific) was used in the SimpliAmp Thermal Cycler (Thermo Scientific). An equal concentration of RNA from each sample was used for the cDNA synthesis. cDNA was diluted 2-fold for all subsequent quantitative PCR (qPCR) reactions. An equal concentration of the diluted cDNA was used along with Power-up SYBR green master mix, to run qPCR reactions on the StepOnePlus Real-time PCR System (4376600, Thermo Scientific). All reactions were run in triplicates and GAPDH was used as housekeeping control. The primer sequences (Sigma-Aldrich) used were as follows:

GAPDH

F5′-CATCACTGCCACCCAGAAGACTG-3′,

R3′-ATGCCAGTGAGCTTCCCGTTCAG-5′

IL-6

F5′-TACCACTTCACAAGTCGGAGGC-3′,

R3′-CTGCAAGTGCATCATCGTTGTTC-5′

IL-10

F5′-CGGGAAGACAATAACTGCACCC-3′,

R3′-CGGTTAGCAGTATGTTGTCCAGC-5′

iNOS

F5′-GAGACAGGGAAGTCTGAAGCAC-3′,

R3′-CCAGCAGTAGTTGCTCCTCTTC-5′

### RNA-seq Analysis

RNA from 78 patient-derived GBM cell cultures was sequenced along with neuroepithelial stem cells (NES) and normal human astrocytes (NHA) as normal controls. Total RNA was extracted using the “AllPrep DNA/RNA/miRNA Universal Kit” (Cat. no 80224, Qiagen) in line with the manufacturer’s protocol. For all samples that passed the threshold values for library preparation, sequencing libraries were prepared from 500 ng total RNA using the TruSeq stranded total RNA library preparation kit with RiboZero Gold treatment (cat# 20020599, Illumina Inc.). Unique dual indexes (cat# 20022371, Illumina Inc.) were used. The library preparation was performed according to the manufacturers’ protocol. Sequencing was performed as paired-end 150bp read length in the NovaSeq 6000 system- S4 flowcell and v1 sequencing chemistry, by the SNP&SEQ Technology Platform in Uppsala, and all samples fulfilled the QC criteria. The RNA-seq data was analyzed using the best practice pipeline nf-core/rnaseq (**DOI:**https://github.com/nf-core/rnaseq/blob/master/docs/output.md). The RNA-seq data was then normalized for gene length and sequencing depth and presented as transcripts per million of 63676 genes. All cell lines passed quality control for the sequencing and were included in the analysis, there was no attrition.

### Microscopy and Imaging

Immunofluorescence images were acquired using a Leica DMi8 inverted fluorescent microscope at 20× magnification. The Leica Application Suite X (LAS X) software was used for image acquisition. Hematoxylin and eosin staining images were acquired with a Zeiss Slide Scanner AxioScan and viewed with Zeiss ZEN 3.1 blue edition software. A standardized algorithm in ImageJ was used to determine the percentage of fluorescently labeled cell number or cell area. Tumor vessel leakage was analyzed using ImageJ software, and IgG leakage was quantified relative to the tumor area. For all quantitative image analyses, a consistent pixel intensity threshold was applied across all images to ensure standardization. Furthermore, cell-specific marker colocalization was assessed based on the overlap and/or spatial proximity of immunolabeling, conducted using the ImageJ plugins: Coloc 2 and Colocalization test.

### Statistical Analysis

Statistical analysis was performed using a one-way Student’s *t*-test, and *P*-values of ≤.05 were considered statistically significant.

## Results

### IL-33 Expression in Human GBM

**Figure 1.**
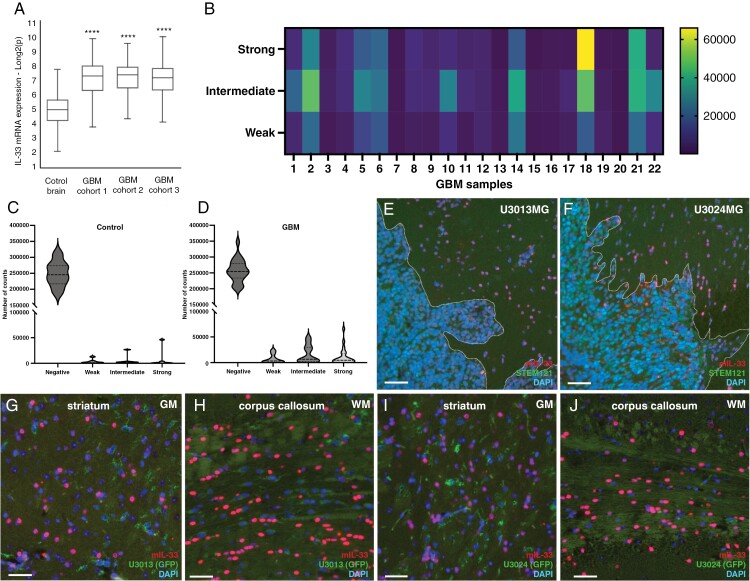
Expression of IL-33 in GBM patients and in xenograft models of glioma. (A) Analysis of publicly available IL-33 mRNA expression data from 3 GBM cohorts^26–28^ compared to control brains.^25^ Expression levels were normalized and presented as fold change. (B) Heat map showing quantification of IL-33 immunolabeling in 22 tumor cores from GBM patients in a tissue microarray (TMA). Automated quantification is presented as staining intensity (weak, intermediate, or strong). (C,D) Violin plots showing quantification of the immunolabelling intensity of IL-33 staining control brain and GBM. Two-way ANOVA of control brain cores versus all GBM cores, *P* = .075. (E, G, H) Expression of murine IL-33 in xenograft mouse models derived by injecting human GBM cells U3013, and (F, I, J) U3024 cells in immunodeficient mouse striatum. The distribution of murine IL-33-expressing cells was examined in cells directly surrounding the tumor mass (E, F, demarcated by dotted line), gray matter of striatum (G, I), and white matter of corpus callosum (H, J). Human cells, stained with STEM121 antibodies. Blue DAPI nucelar stain. Representative data from three mice for each GBM cell line injected. Data are presented as mean ± SD, ****P < .0001. Scale bar 50 µm.

To investigate the expression of IL-33 in brain tumors, we first analyzed publicly available data for IL-33 mRNA expression using three GBM cohorts^26–28^ and observed an increase in IL-33 mRNA when compared to control brain^25^ (Figure 1A). Next, we assessed IL-33 immunostaining of a tissue microarray (TMA) with GBM tumor cores (*n* = 22) and control brain tissues (*n* = 7; Supplementary Figure S1). Automated quantification reveals the proportion of negative, weak, intermediate, or strong IL-33 staining in each tumor core (Figure 1B-D, Supplementary Figure S1A). The heatmap in Figure 1B, shows that GBM cores display heterogenous expression of IL-33 between patients (Figure 1B). The percentage of IL-33-positive cells in each category was calculated and compared to non-tumor brains. Violin plots in Figure 1C-D show a tendency for higher expression of IL-33 protein in GBM tissue versus control brain, but not statistically significant (two-way ANOVA of control brain cores versus GBM cores, *P* = .075).

Previous studies have suggested a correlation between high IL-33 expression and poorer survival for GBM patients.^33,34^ We therefore performed Kaplan–Meier analysis on the overall survival data of the TCGA GBM cohort (The Cancer Genome Atlas, www.cancer.gov). By dividing the dataset samples into two groups based on the IL-33 expression (lowest vs. highest quartile), a weak trend for shorter survival is observed for patients with high expression (Supplementary Figure S2A), but the difference is not significant. We also performed a Cox Hazard ratio analysis of the TCGA cohort, and the Hegi cohort (GSE7696), separating overall survival and progression-free survival based on IL-33 mRNA expression levels. We find no significant differences in survival between the groups (Supplementary Figure S2B).

### ST2 Expression in Human GBM

To investigate ST2 (also known as IL1RL1) expression in brain tumors, we analyzed publicly available GBM datasets for ST2 mRNA expression from GBM cohorts^26–28^ and observed a reduction of ST2 levels in GBM patients, compared to the control brain^25^ (Supplementary Figure S2C). Furthermore, patients in the TCGA database exhibiting high versus low expression of ST2 did not show any difference in overall survival (Supplementary Figure S2D). Additionally, we investigated the expression of ST2 in 78 patient-derived GBM cell lines, maintained under stem cell culture conditions,^31^ and detect very low ST2 expression (Supplementary Figure S2E). In comparison, the expression of IL-33 in the GBM cells varied (Supplementary Figure S2F), in line with the varying IL-33 protein expression in the TMA (Figure 1A).

### Widespread IL-33 Expression in the Mouse Tumor Microenvironment

Next, we focused on IL-33 in the tumor microenvironment, using xenograft mouse models where GBM cells from 2 different patients were injected into the striatum of immunodeficient mice (Supplementary Figure S3A, D). Brain tumor sections were subjected to immunohistochemistry with murine-specific IL-33 antibodies to detect host-derived IL-33. We find that IL-33-positive cells mainly reside outside of the tumor bulk and are localized both close to the tumor (Figure 1E, F) and further away in the ipsilateral hemisphere (Supplementary Figure S3B, E), and contralateral hemisphere (Supplementary Figure S3C, F). Both gray (Figure 1G, I) and white matter (Figure 1H, J) tracts stain positive for IL-33. Since the majority of IL-33 staining was detected outside the tumor core, we performed immunolabeling for IL-33 in U3013 and U3024 cells cultured in vitro to evaluate inherent IL-33 levels in the grafted GBM cells. We observe weak immunostaining (Supplementary Figure S4A-B), and in addition, GBM cells display low z-scores for IL-33 mRNA, although higher than those of NHA (Supplementary Figure S4C).

### Oligodendrocytes are the Major Source of IL-33 in the Murine Glioma Microenvironment

**Figure 2:**
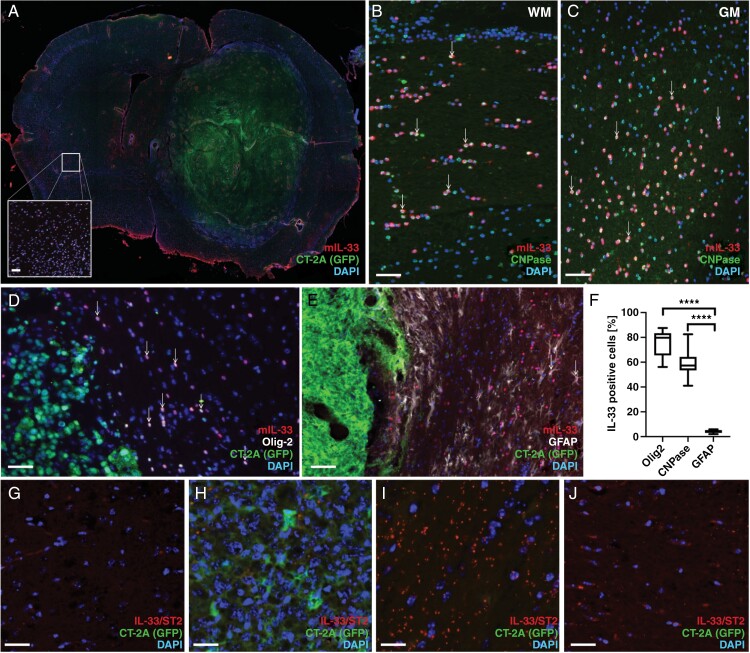
IL-33 expression in the tumor microenvironment of mouse glioma models. (A) Immunohistochemical analysis showing IL-33 expression in the mouse brain with CT-2A tumor cells. IL-33 expression in the contralateral hemisphere (A, insert). (B-C) Co-expression of IL-33 and CNPase in the white matter of the corpus callosum (WM, B), and in the gray matter of the cortex (GM, C). (D) Co-localization of IL-33 with Olig2 or (E) GFAP. (F) Quantification of co-localization of IL-33 with Olig2, GFAP or CNPase in the tumor brain. (G-J) Proximity ligation assay detects protein complexes formed by IL-33 and ST2 in CT-2A glioma. (G) control brain, (H) tumor core, (I) area surrounding the tumor, and (J) contralateral hemisphere. Results are representative of three biological replicates with 6 mice per group in each repeat. Data are presented as mean ± SD, ****P < .0001. Scale bar 50 µm.

We and others have previously shown that in neurodevelopment and neuroinflammation, astrocytes and oligodendrocytes, but not neurons or microglia, express IL-33.^21,22,35,36^ To investigate if this is the case also in glioma, 2 syngeneic mouse models were used. Mouse glioma cells, CT-2A^37^ or GL261,^38^ were injected into the striatum of C57BL/6 mice. Tumor progression was monitored daily by assessing symptoms such as kyphotic posture, lethargy, or weight loss exceeding 10%. Mice were euthanized when symptom scores reached the threshold specified by the Ethical Review permit. Whole brain sections were used for immunohistochemical staining with antibodies to IL-33. Similarly to what was observed in our xenograft models, in both the CT-2A (Figure 2A) and GL261 (Supplementary Figure S5A-C) immunocompetent models, IL-33 expression is located mostly outside the tumor bulk. Neither of these mouse glioma cell lines express detectable levels of IL-33 when cultured in vitro (Supplementary Figure S5D-E).

To identify the IL-33-expressing cells, we performed double-labeling immunofluorescence. Using an antibody against CNPase, a marker for oligodendrocytes, we observe IL-33 and CNPase co-expressing cells both in white matter tracts (Figure 2B) and in gray matter regions (Figure 2C). Antibodies to OLIG2, a marker of oligodendrocytes and oligodendrocyte progenitor cells, also co-label IL-33 positive cells in the tumor microenvironment (Figure 2D). Additionally, staining for GFAP reveals astrocytes which were double-positive for IL-33 (Figure 2E). Analysis of coronal sections across the tumor-bearing brain showed that a majority of IL-33-positive cells (**≥**80%) also co-express OLIG2, and more than half (around 60%) co-express CNPase (Figure 2F) while co-labeling with GFAP is seen in 5% of the IL-33-positive cells (Figure 2F). These results indicate that the oligodendrocyte lineage is the primary cellular source of IL-33 in the glioma microenvironment.

### Presence of IL-33/ST2 Complexes Outside of the Tumor Core

To investigate if the glia-derived IL-33 interacts with its receptor ST2 in CT-2A-derived tumors, we used an antibody-based dual recognition proximity ligation assay to visualize protein complexes formed by IL-33 and ST2. As shown above, almost no IL-33 is detected in the intact brain and complexes of IL-33 and ST2 are scarce (Figure 2G). Very few complexes are visible within the tumor core (Figure 2H). In contrast, in the area surrounding the tumor (Figure 2I) we detect protein complexes, also in the contralateral hemisphere (Figure 2J). Our data thus show that the host brain responds to tumor growth by upregulating IL-33, which can be found as complexes with its receptor, ST2, both close to the tumor, and far from the tumor site, suggestive of an inflammatory response.

### *Increased Glioma Aggressiveness in* ST2*-Deficient Mice*

**Figure 3.**
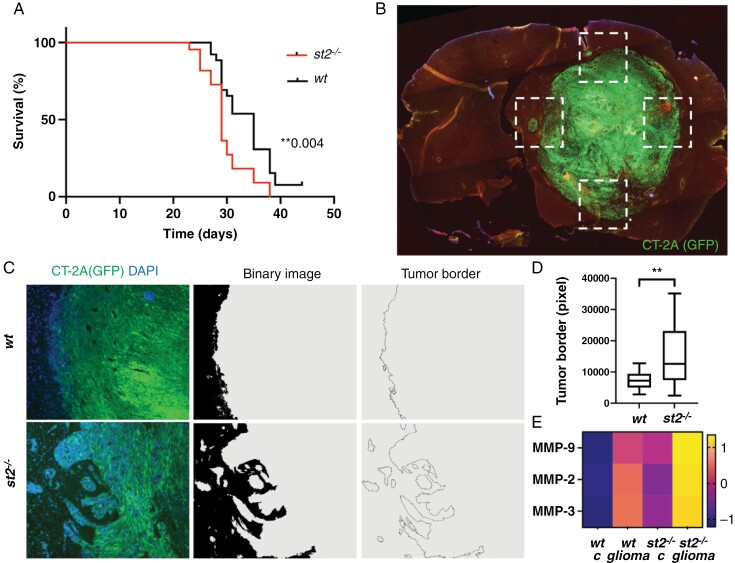
Increased glioma aggressiveness in ST2-deficient mice. (A) Kaplan–Meier analysis showing shorter survival times in ST2-deficient mice (*n* = 30) with CT-2A glioma, compared to *wt* mice (*n* = 30). (B-C) Image processing to measure the length of the tumor border in ST2-deficient and *wt* mice. (D) Quantification of the tumor border length, with longer borders observed in ST2-deficient mice compared to *wt*. (E) Increased matrix metalloproteinases (MMP2, MMP3, and MMP9) in the serum of ST2-deficient mice, further enhanced in ST2-deficient mice with tumors. Scale bars: 1 mm (B) and 50 µm (C). (B-D) Results are representative of three biological replicates with 10 mice per group in each repeat. Data are presented as mean ± SD. Scale bar 50 µm.

To assess the role of the IL-33/ST2 axis in glioma growth in vivo, we injected CT-2A glioma cells into the brains of ST2-deficient mice and monitored their tumor burden and survival outcome, compared to that of wild-type (*wt*) mice. Mice of the 2 genotypes started to develop tumors approximately 7 days after injection and were sacrificed when the humane endpoint was met, around 25–30 days after tumor cell grafting. Kaplan–Meier analysis revealed that ST2-deficient mice exhibit shorter survival times compared to *wt* mice (Figure 3A). To gain insight into the mechanisms underlying the difference in survival between the 2 groups, we analyzed whole-brain sections from tumor-bearing mice. We observe no differences in the amounts of IL-33-expressing oligodendrocytes (Supplementary Figure S6A-D) or astrocytes (Supplementary Figure S6E-H) in the tumor microenvironment between the 2 genotypes. This indicates the absence of a regulatory feedback loop to promote or decrease the proportion of IL-33 positive cells when its receptor is absent.

Interestingly, however, we observed that in ST2-deficient brains, the interface between the tumor and host brain exhibits a more irregular pattern with numerous protruding lobes of tumor cells, suggestive of a more invasive phenotype (Supplementary Figures S7 and S8). To investigate the characteristics of the invasive front, we analyzed the tumor/brain interface in four regions per brain (Figure 3B) and developed a sequence for digital image processing that allowed us to visualize and quantify the tumor border area by measuring the length of the tumor border (Figure 3C). Our analysis reveals that tumor sections derived from ST2-deficient mice exhibit longer tumor borders, compared to *wt* mice (Figure 3D), indicative of a more invasive tumor phenotype. In line with this, a higher baseline presence of matrix metalloproteinases MMP-2, MMP-3, and MMP-9 is observed in the serum of ST2-deficient mice compared to *wt* mice (Figure 3E). This difference is further enhanced upon tumor formation (Figure 3E). These results suggest that the lack of IL-33/ST2 signaling facilitates CT-2A tumor invasion, potentially through enhanced expression of MMPs.

### Aggravated Tumor Vasculature in the Absence of ST2

**Figure 4.**
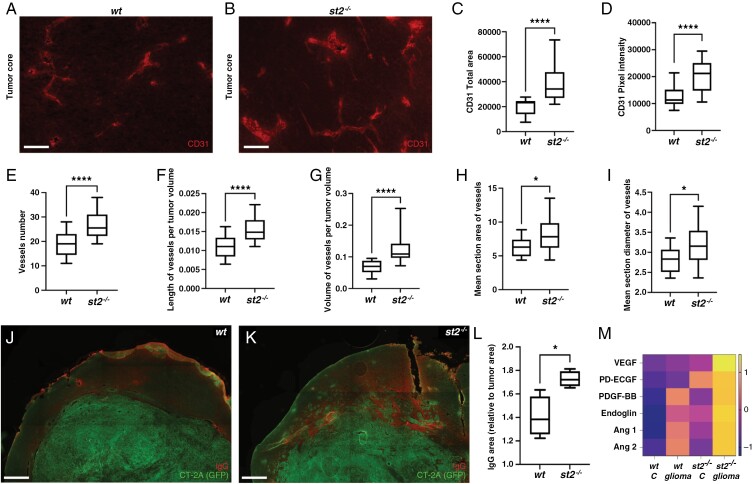
Aggravated tumor vasculature in the absence of ST2 in mouse glioma. (A-B) Immunofluorescence staining showing increased CD31 expression and vascular density in gliomas from ST2-deficient mice. In the absence of *st2*, CD31 expression increases, as evidenced by (C) area marked by CD31 antibodies and (D) intensity of the staining close to the tumor border. There is a higher number of vessels (E), extended vessel length (F), and larger vessel volume (G). (H) Vessels in *st2*^*-/-*^ mice are characterized by a greater cross-sectional area and (I) an increased diameter. (J-L) IgG labeling reveals that ST2-deficient glioma-bearing mice exhibit more leakage than *wt* mice in close proximity of the tumor border (M) Elevated levels of proteins associated with angiogenesis in serum of ST2-deficient mice with brain tumors, compared to control mice, and further enhanced in *st2*^*-/-*^ mice with glioma. Results are representative of three biological replicates with 10 mice per group in each repeat. Scale Bars: 50 µm (A, B) and 500 µm (J, K).

Next, we explored the impact of ST2 on tumor angiogenesis, since aberrant vessel formation is a hallmark of malignant glioma. Immunofluorescence staining of brain tumor sections using an antibody to endothelial cell marker CD31 reveals notable differences between ST2-deficient and *wt* mice (Figure 4A-B). Tumors growing in the absence of ST2 exhibit a higher CD31 positive vessel area (Figure 4C), and an increased intensity of the CD31 antibody staining within these vessels (Figure 4D). Vessel number (Figure 4E), length (Figure 4F), and volume (Figure 4G) are increased in ST2-deficient mice, as well as their cross-section area (Figure 4H) and vessel diameter (Figure 4I). This indicates that the lack of ST2 receptor signaling promoted glioma-associated tumor angiogenesis.

To assess the functionality of tumor blood vessels, we performed fluorescent labeling of CT-2A-derived tumor sections with anti-IgG antibodies, to detect vascular leakage of high molecular weight molecules such as endogenous IgG. Notably, we find that ST2-deficient glioma-bearing mice exhibit more leakage than *wt* mice in the proximity of the tumor border (Figure 4J-K). Quantification of the stained area confirms an enhanced IgG extravasation in the absence of ST2 (Figure 4L), indicative of increased vessel permeability.

In line with this, significantly elevated levels of angiogenesis-associated proteins are found in the serum of tumor-bearing ST2-deficient mice, compared to control mice. (Figure 4M). Among these are VEGF, which plays a pivotal role in tumor angiogenesis and vascular permeability, as well as PD-ECGF and PDGF-BB.^39^ Furthermore, angiopoietins 1 and 2, which exert crucial roles in the angiogenic switch during tumor progression are also increased. Taken together, our findings suggest that ST2 deficiency dysregulates vascular remodeling, potentially leading to the establishment of a more extensive, and leaky, glioma-associated vasculature.

### ST2 Deficiency Results in Suppressed Immune Responses in CT-2A Glioma

**Figure 5.**
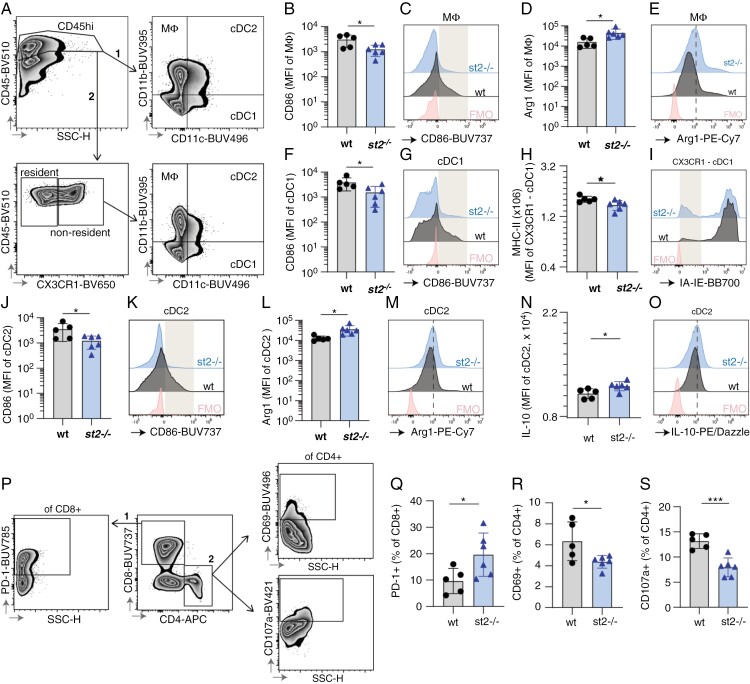
Absence of ST2 results in suppressed immune response in CT-2A gliomas. Flow cytometry data of immune cell profiles in CT-2A brain tumors from *wt* and *st2*^*-/-*^ mice. (A) Gating strategy used to identify innate myeloid populations in the brain of CT2A-bearing wt and *st2*^*-/-*^ mice by flow cytometry, namely CD11b^+^CD11c^-^ macrophages (MΦ), CD11b^-^CD11c^+^ cDC type 1 (cDC1) and CD11b^+^CD11c^+^ cDC type 2 (cDC2). The overall macrophage, cDC1, and cDC2 populations were identified by gating on CD45^high^ cells, followed by gating based on CD11b and CD11c expression. Alternatively, non-tissue resident cDC1s were identified as CD45^high^CX3CR1^-^CD11b^-^CD11c^+^. Macrophages (CD11b + CD11c-) from *st2*^*-/-*^ mice have reduced CD86 expression (B,C) and increased Arg1 levels (D, E), indicating suppressed activity. The gray box in (D) highlights a population of CD86^hi^ cells that were underrepresented in *st2*-/- mice. (D,E) The dotted line in (E) indicates increased expression of Arg1 in *st2*-/- mice. Type 1 conventional dendritic cells (CD11b-CD11c + cDC1s) in *st2*^*-/-*^ mice show lower CD86 (F, G), and nonresident CX3CR1- cDC1s display reduced MHC-II expression (H, I), crucial for T cell activation. The gray box in (G) highlights a population of CD86^hi^ cells that were underrepresented in *st2*-/- mice while the gray box in (I) highlights a population of MHC-II^-^ cells that were overrepresented in *st2*-/- mice. Similarly, type 2 cDCs (CD11b + CD11c + cDC2s) display decreased CD86 (J, K) with heightened Arg1 (L, M) and IL-10 production (N, O), suggesting immune inhibition. The gray box in (K) and (O) highlights a population of CD86^hi^ and IL-10^hi^ cells that were underrepresented in *st2*-/- mice. The dotted line in (M) indicates increased expression of Arg1 in *st2*-/- mice. (P) Gating strategy used to produce data shown in panels Q-S. The two major T cell subpopulations were separated based on their CD8 and CD4 expression. CD8^+^PD1^+^, as well as CD4^+^CD69^+^ and CD4^+^CD107a^+^ T cells, were identified using FMO-based gates. An increase in exhausted CD8 + PD1 + T cells (Q) and a decrease in activated (CD69+) (R) and cytotoxic (CD107a+) (S) CD4 + T cells in *st2*^*-/-*^ mice indicate T cell suppression. Results are representative of three biological replicates with 10 mice per group in each repeat.

The tumor microenvironment is crucial for shaping the immune response against cancer. To assess whether the anti-tumor immune response was affected by the absence of ST2, we performed flow cytometry analysis of CD45^+^ cells isolated from CT-2A tumors implanted in *wt* or ST2-deficient mice, with a special focus on macrophages (MΦ), and conventional DCs (Figure 5A, Supplementary Figure S9), or CD4 and CD8 positive lymphocytes (Figure 5P, Supplementary Fig S10). In ST2-deficient mice, tumor-infiltrating CD11b^+^CD11c^-^ macrophages, exhibit decreased expression of the co-stimulatory receptor CD86 (Figure 5B-C) and produce increased levels of the immunosuppressive enzyme Arg1 (Figure 5D-E). Their proportion of CD45^+^ cells is however not altered (Supplementary Figure S11A). Conventional dendritic cells (cDCs) also show an impaired activation in the absence of ST2. Indeed, in ST2-deficient mice CD11b^-^CD11c^+^ type 1 cDCs (cDC1) exhibit lower levels of CD86 (Figure 5F-G), and nonresident CX3CR1^-^CD11b^-^CD11c^+^ cDC1s display a decreased expression of the antigen-presenting molecule MHC-II (Figure 5H-I) in ST2-deficient mice. Similarly, CD11b^+^CD11c^+^ type 2 cDCs (cDC2) show decreased CD86 expression (Figure 5J-K) and increased production of Arg1 in tumors implanted in mice lacking ST2 (Figure 5L-M). Moreover, a small but significant increase in the production of IL-10 is also observed in cDC2s in the absence of ST2 (Figure 5N-O). No difference in the overall proportion of macrophages, cDC1s, or cDC2s (% of CD45^high^ immune cells) was observed (Supplementary Figure 11B-C).

Consistent with the immunosuppressed myeloid compartment, an increased percentage of CD8^+^ T-cells expressing PD1, a marker often associated with T-cell suppression, is observed in CT-2A tumors implanted in ST2-deficient mice (Figure 5Q, Supplementary Figure S11F), accompanied by decreased proportions of activated (CD69^+^; Figure 5R; Supplementary Figure S11H) and cytotoxic (CD107a^+^) CD4^+^ T cells (Figure 5S; Supplementary Figure S11G). The proportion of CD8 + and CD4 + T cells is unaltered (Supplementary Figure S11 D-E). Finally, gene expression analysis of tumor tissues reveals that ST2-deficient mice exhibited increased overall levels of IL-4 and IL-6 (cytokines associated with the Th2-immune response, and with the accumulation of myeloid-derived suppressor cells^40^), as well as of the immunosuppressive cytokine IL-10, while iNOS remain unaltered (Supplementary Figure S12).

Altogether, this data suggests that the absence of ST2 negatively affects the anti-tumor immune response in CT-2A glioma and leads to suppression of the myeloid and lymphoid immune response against the tumor.

## Discussion

Cytokines and other inflammatory mediators released by cells in the tumor microenvironment have profound effects on tumor progression. IL-33 is an alarmin secreted by glial cells, which binds to the ST2 receptor expressed on immune cells. IL-33 and has gained attention for its contribution to tumor progression,^41^ and has also been shown to have both pro- and anti-tumorigenic effects (reviewed by Robbins^19^) in a context-dependent manner. Indeed, although the role of the IL-33 and ST2 interplay in cancer has not been fully elucidated, it appears that IL-33 signaling can have both beneficial effects in certain experimental tumors,^42,43^ while in other studies it has been reported that IL-33 contributes to GBM tumorigenesis.^17,33,34,44^ IL-33 could thus be associated with tumor progression,^7,9,34^ or, alternatively, part of an alarmin response to a growing tumor. Both of these processes might also operate simultaneously, depending on whether IL-33 is expressed in GBM cells or not.^19^

In glioma, non-tumor cells within the tumor microenvironment, such as macrophages and microglia, play critical roles that influence clinical outcomes. Over time, a shift occurs within the tumor, with a decreasing proportion of malignant cells and an increasing contribution of non-tumor cells to the tumor’s cellular composition.^45^ It is well known that oligodendrocytes and oligodendrocyte progenitor cells play a role in CNS injury (reviewed in^46^). Here, we demonstrate that IL-33 expression is primarily restricted to the oligodendrocyte lineage in mouse models of glioma. Glioma-associated oligodendrocyte progenitor cells are known to facilitate neovascularization by promoting endothelial sprouting and tubule formation.^47^

Using ST2-deficient mice as hosts for transplanted glioma, we demonstrate that lack of the ST2 receptor significantly worsens survival and promotes an invasive tumor phenotype. ST2-deficient mice exhibited extensive and irregular tumor borders, denoting increased invasiveness. This change was mirrored by a rise in serum MMPs, known for their crucial roles in promoting glioma invasiveness by facilitating the degradation of the extracellular matrix.^48^ This implies that ST2 deficiency could lead to a more aggressive tumor phenotype by facilitating tumor invasion and the formation of a more supportive environment for glioma growth.

IL-33 has been reported to increase the proliferation and migration of endothelial cells, and induce angiogenesis in vitro and in vivo,^49^ and through the ST2 receptor, it has been shown to regulate blood vessel remodeling in various types of injuries. However, deletion of the ST2 receptor has also been shown to favor angiogenesis.^50^ Here, we observe increased numbers and density of aberrant blood vessels in gliomas implanted in ST2-deficient mice, in association with increased levels of VEGF in the serum, a crucial factor known for promoting tumor vascularization and supporting tumor growth.^39^ This data indicates that ST2 deficiency promotes an angiogenic microenvironment, associated with the release of MMPs and angiogenic factors, which favors vascular abnormalities in glioma.

IL-33 has the ability to attract and stimulate various immune cell populations within the glioma microenvironment, including CD8 + T cells, CD4 + T cells, dendritic cells (DCs), macrophages, and regulatory T cells (Tregs).^17,51–53^ We demonstrate that the absence of ST2 in the glioma microenvironment negatively impacts both the CD4^+^ and CD8^+^ T cell populations, by decreasing their activation or increasing their expression of checkpoint molecule PD1, respectively. Additionally, IL-33 was reported to promote the maturation and antigen-presenting capacity of DCs,^54,55^ facilitating the activation of T-cell responses against glioma antigens. Our data show that DC activation was impaired in the absence of ST2, which together with expression of Arg1 on macrophages may explain the lower level of markers of activation and cytotoxicity on CD4 T cells.

In summary, our study demonstrates that the absence of the IL-33 receptor ST2 is associated with increased tumor invasion, vascular abnormalization, and immunosuppression in murine glioma models where IL-33 is predominantly expressed by oligodendrocytes in the tumor environment. This suggests that glia-derived IL-33 participates in orchestrating the brain’s anti-tumor response in an ST2-dependent manner.

## Supplementary Material

vdaf010_suppl_Supplementary_Material

## Data Availability

GBM cell cultures can be obtained pending a Material Transfer Agreement upon contact with the Human Glioma Cell Culture biobank at mail@hgcc.se. Information and requests for other resources and reagents should be directed to the corresponding author.
