# Supplementary material for: Lack of ST2 aggravates glioma invasiveness, vascular abnormality, and immune suppression
Source: Neurooncol Adv. 2025 Jan 27;7(1):vdaf010. doi: 10.1093/noajnl/vdaf010 (PMC11808570; doi:10.1093/noajnl/vdaf010)
Supplement: vdaf010_suppl_Supplementary_Material [file vdaf010_suppl_supplementary_material.docx]

**Supplementary figures**

**Figure S1**


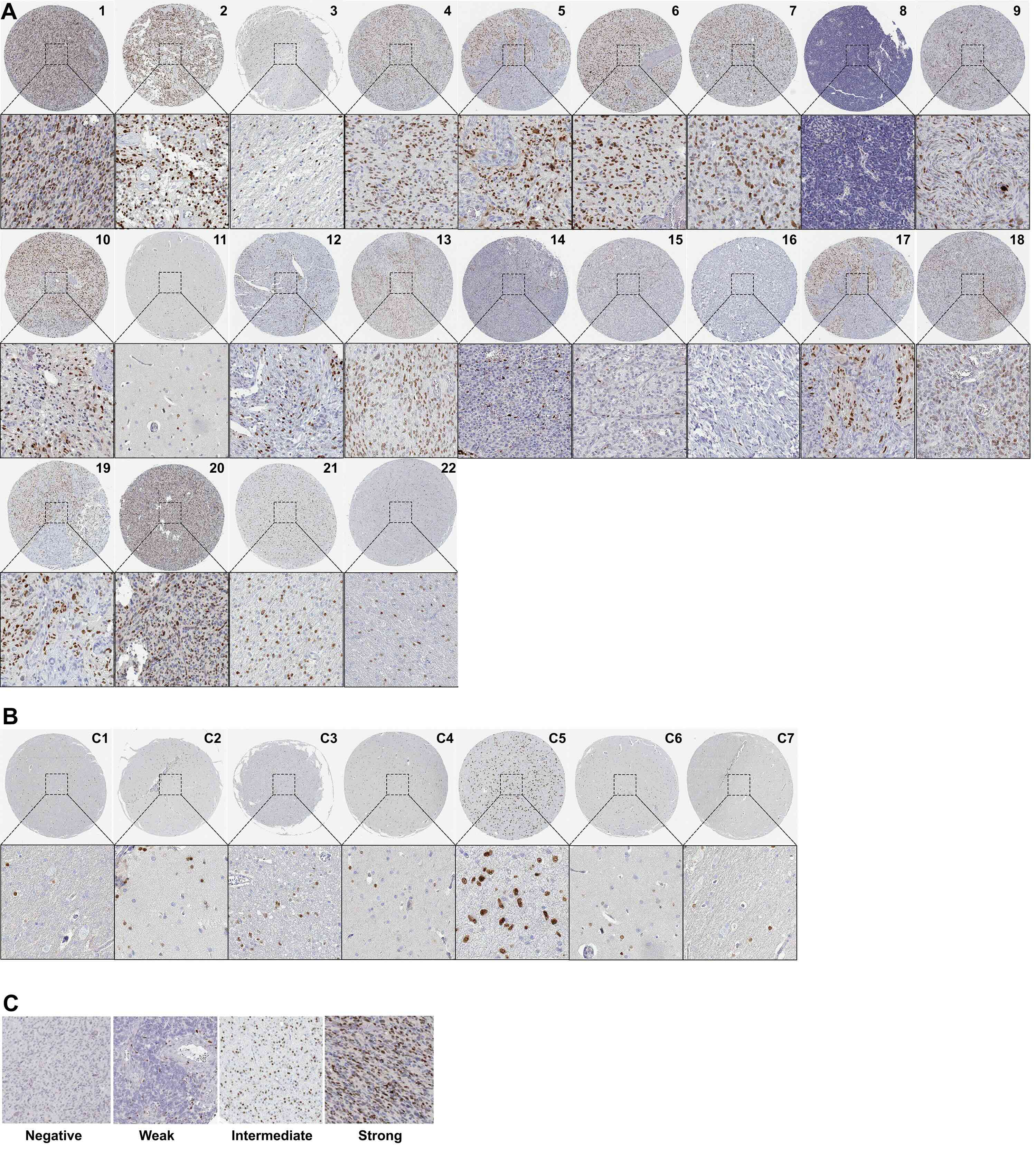


**Human GBM and non-tumor tissue microarray (TMA) stained with IL-33 antibodies**

Figure showing 22 samples from GBM patients of a tumor microarray (TMA) immunolabeled for IL-33, shown in the form of a core overview and an inset with magnification for each sample. B) Examples of immunolabeling for IL-33 categorized as negative, weak, intermediate, and strong by automatic quantification.

**Figure S2
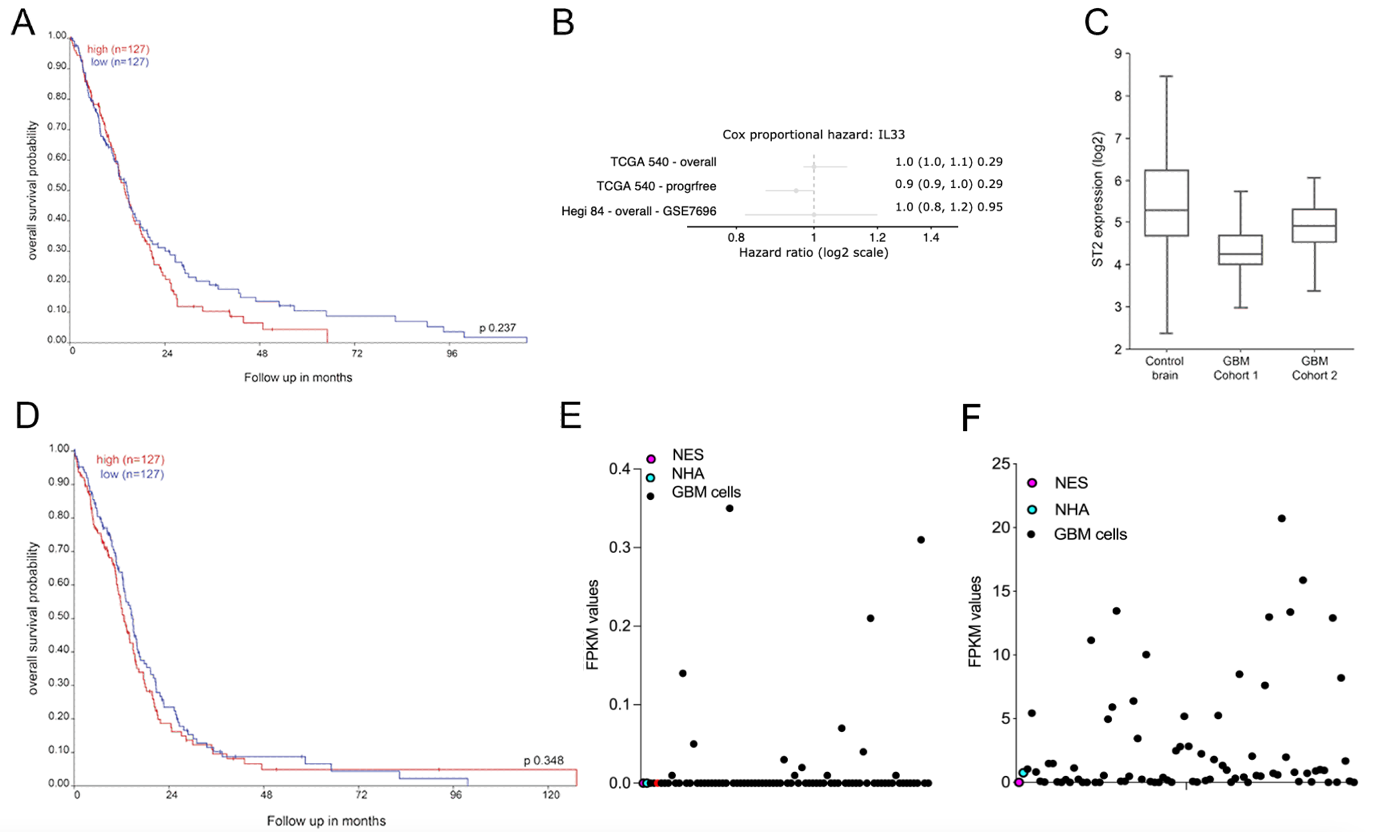
**

**IL-33 and ST2 expression in human GBM and GBM cells**

A) Kaplan Meier curve for overall survival of the TCGA GBM patients was plotted using the first versus last quartile values of IL33 expression as cut-off. No significant survival difference is observed between the groups.

B) Cox Hazard ratio analysis of the TCGA cohort, and the Hegi cohort (GSE7696) separating overall survival and progression-free survival, for IL33, based on expression levels

C) Expression levels of ST2 mRNA from two publicly available GBM cohorts [24-25] compared to control [27] brains. ST2 expression in GBM was lower than normal brain (ANOVA p-value 5.82e-08).

D) Kaplan Meier curve for overall survival of the TCGA GBM patients was plotted using the first versus last quartile values of ST2 expression as cut-off. No significant survival difference is observed between the groups.

E) Expression (RNA seq) for ST2 (IL1RL1) in our cohort of human GBM-derived cell lines (n=78) compared to normal human astrocytes (NHA, blue) and neuroepithelial stem cells (NES, orange) as d non-tumor control. Over 90% of the cells have below detectable levels of ST2, similar to the control cells.

F) Expression (RNA seq) of IL-33 in our cohort of human GBM-derived cell lines (n=78) compared to normal human astrocytes (NHA, blue) and neuroepithelial stem cells (NES, orange) as non-tumor control.

**Figure S3**

**
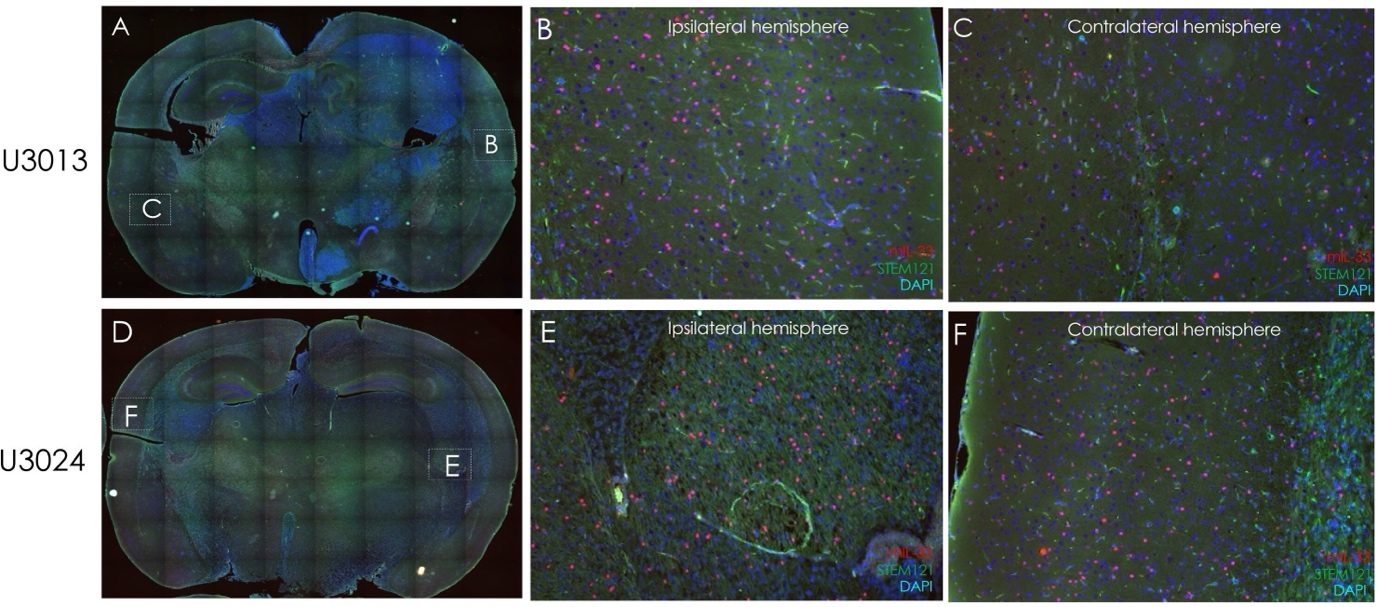
**

**Xenografts of patient-derived GBM cells in *Foxn1^-/-^* nude mice.**

Immunohistochemistry staining with murine-specific IL-33 antibodies reveals IL-33-positive cells (red) predominantly outside of the tumor mass in immune-deficient mouse brains grafted with A-C) U3013MG (n=3) or D-F) U3024MG (n=3), within the same hemisphere (B, E) as well as the contralateral hemisphere (C, F). STEM21, green depicts human cells, blue DAPI nuclear stain.

**Figure S4
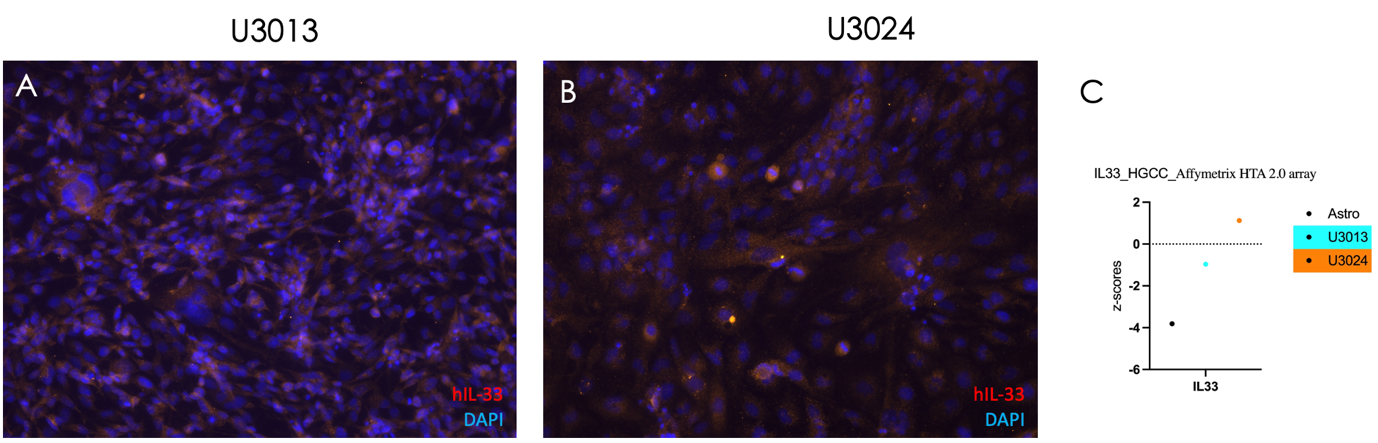
**

**Low IL-33 expression in patient-derived GBM cell cultures used for xenografts**

Immunocytochemistry staining with IL-33 antibodies shows weak expression of IL-33 (red) in cultures of human GBM cells U3013 (A) and U3024(B). Blue DAPI nuclear stain.

C) z-scores of IL-33 mRNA expression in U3013 and U3024 cells, compared to normal human astrocytes (astro) determined by Affymetrix HTA 2.0 array.

**Figure S5**

**
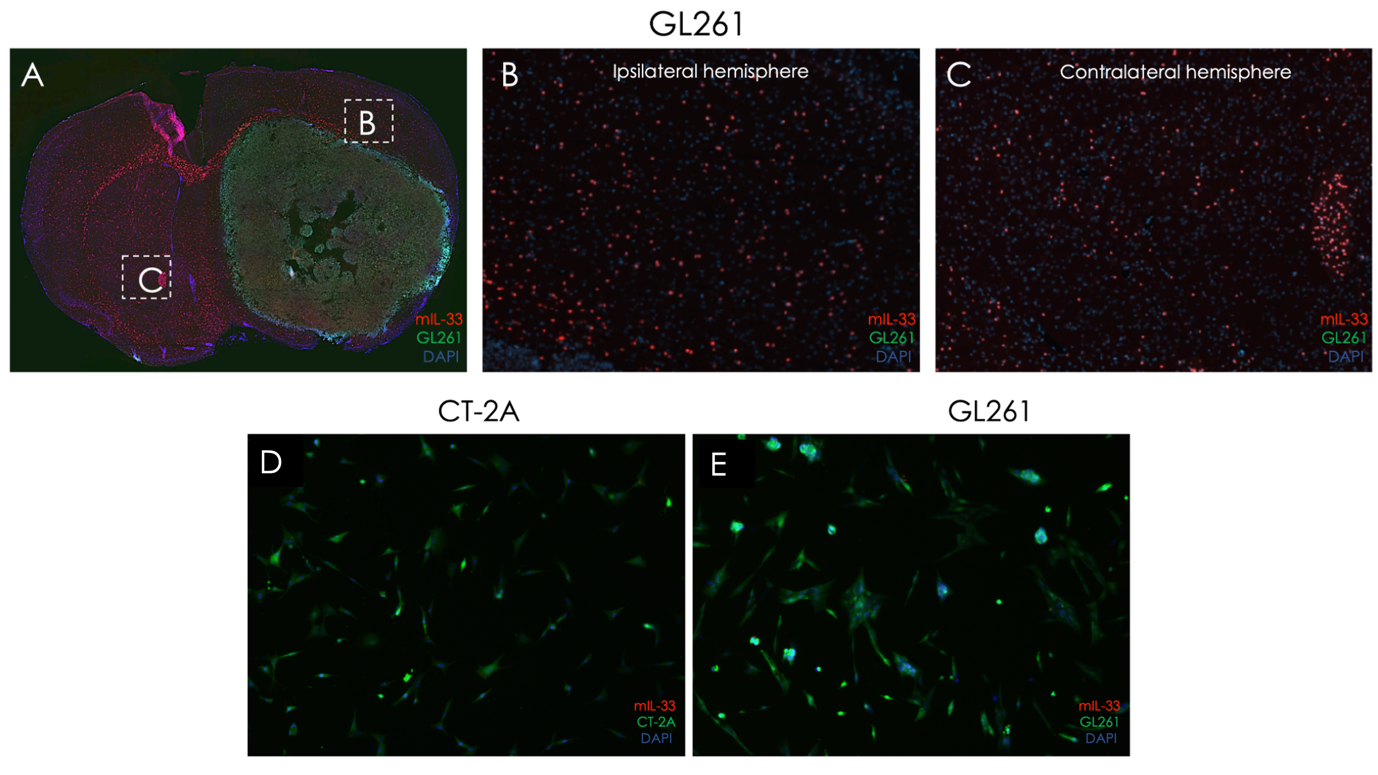
**

**IL-33 distribution in brains of C57BL/6 mice transplanted with GL261 glioma cells.**

A-C) Immunohistochemical staining with IL-33 antibodies reveals extensive IL-33 expression (red) in areas beyond the tumor mass in GL261 cell-injected mouse brain, with localization within the same hemisphere (B) and the contralateral hemisphere (C). CT-2A and GL261 cells were labelled by their GFP expression, Blue DAPI nuclear stain. Representative data from two biological repeats with 10 fields analyzed for each brain.

D-E) Immunocytochemistry staining IL-33 antibodies fails to detect IL-33 in cultures of mouse glioma cell lines CT-2A (D) and GL261 (E). ). CT-2A and GL261 cells were labelled by their GFP expression, Blue DAPI nuclear stain. Representative data from two biological repeats with 5 fields analyzed for each cell culture.

**Figure S6**

**
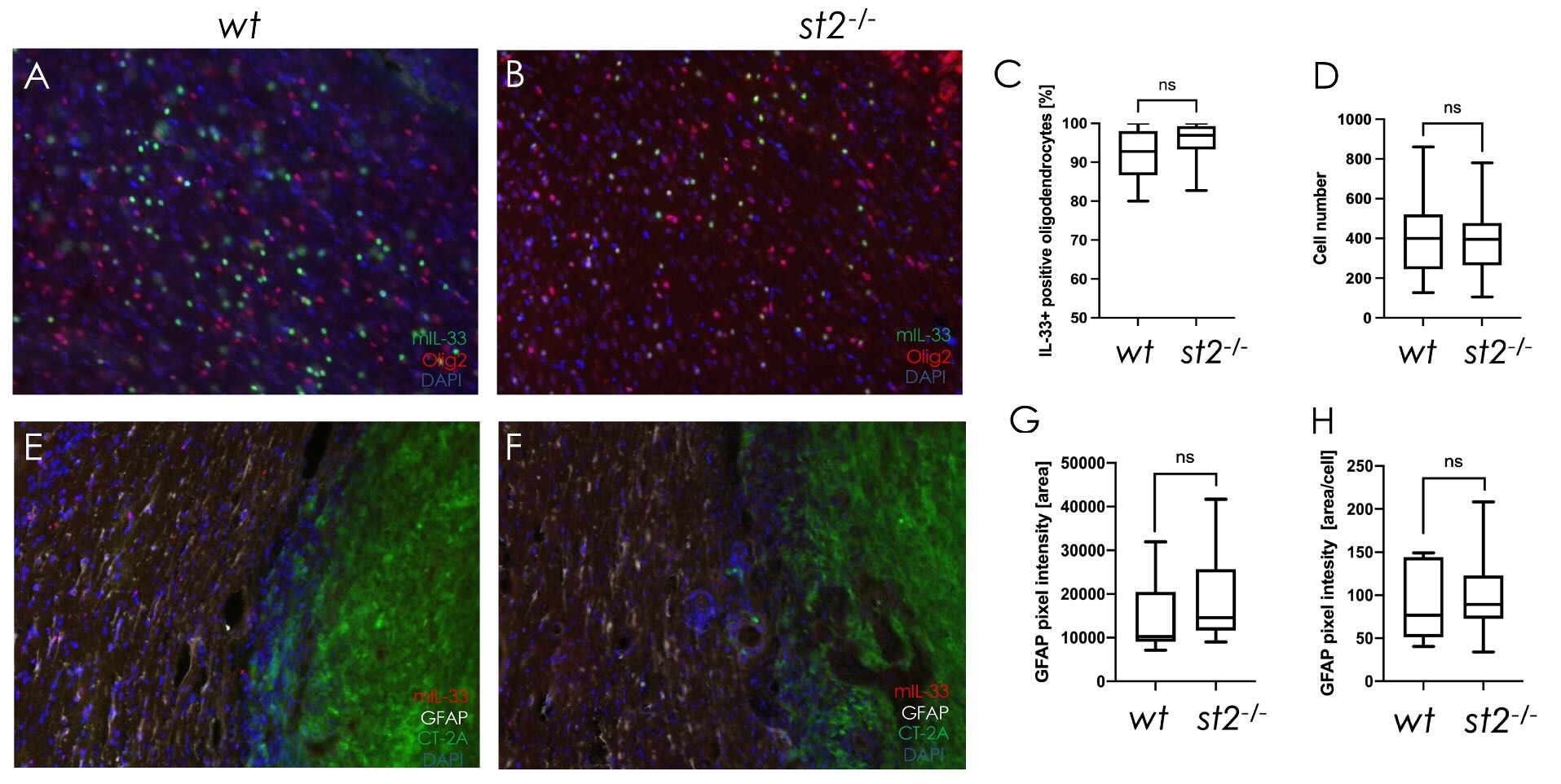
**

**Similar IL-33 expression in glia cells in *st2^-/-^* and *wt* brains transplanted with CT-2A glioma cells**

A-D) No difference was observed in IL-33 expression (green A-B, red E-F) of oligodendrocytes (red) or E-H) astrocytes white) in the brains of *st2^-/-^* and *wt* mice that developed glioma after grafting CT-2A cells (green). Results are representative of three biological replicates using 8 mice per group. 10 fields were analyzed for each brain. Data are presented as mean± SD.

**Figure S7**

**
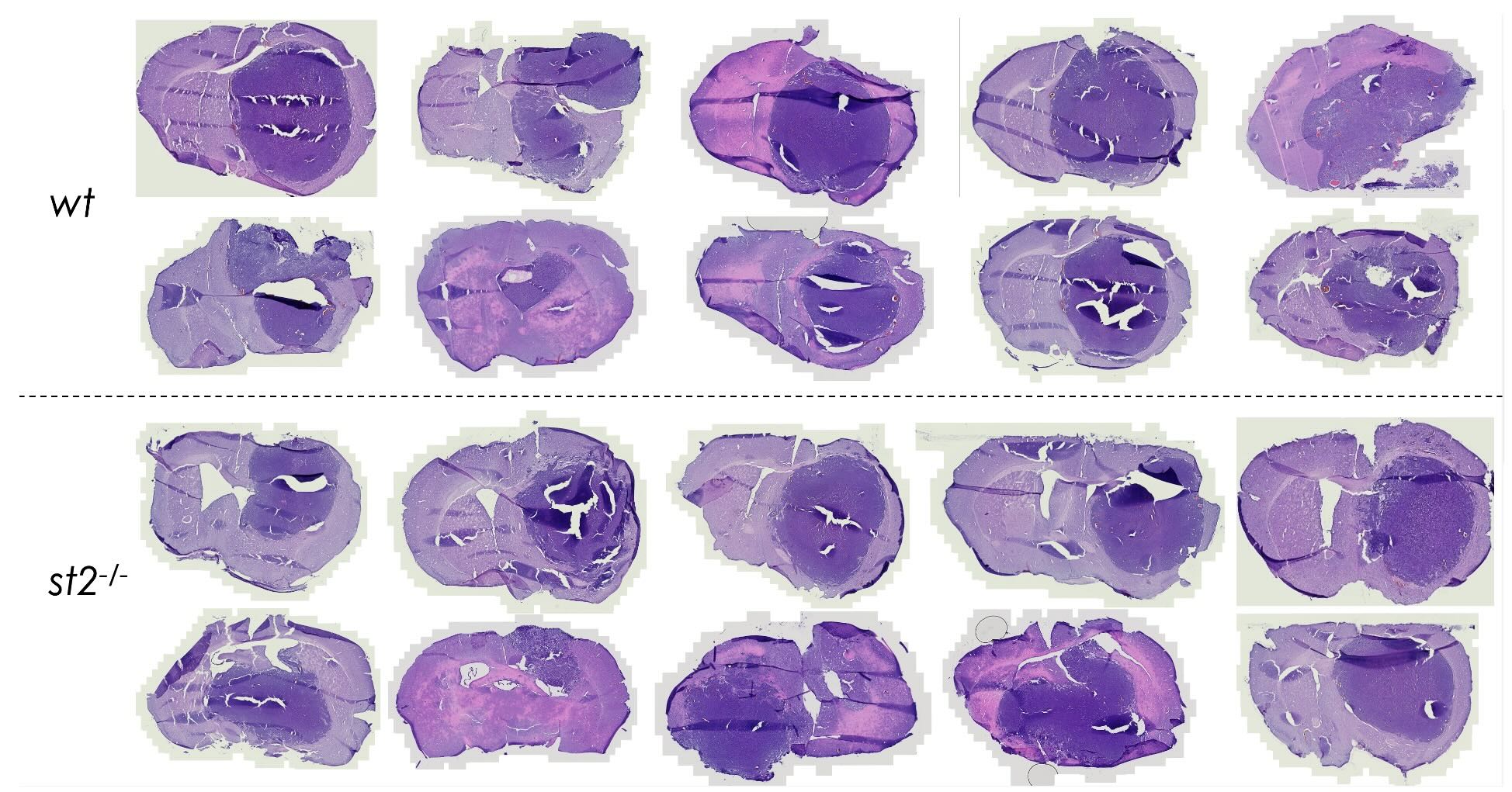
**

**Hematoxylin/eosin (H/E) staining of CT-2A-grafted to C57/Bl6 mouse brains**

H/E staining of sections through mouse brains with tumors after injecting C2-2A cells

*st2^-/-^* (n=10) and *wt* (n=10) mice. Representative data from three repeats.

**Figure S8**

**
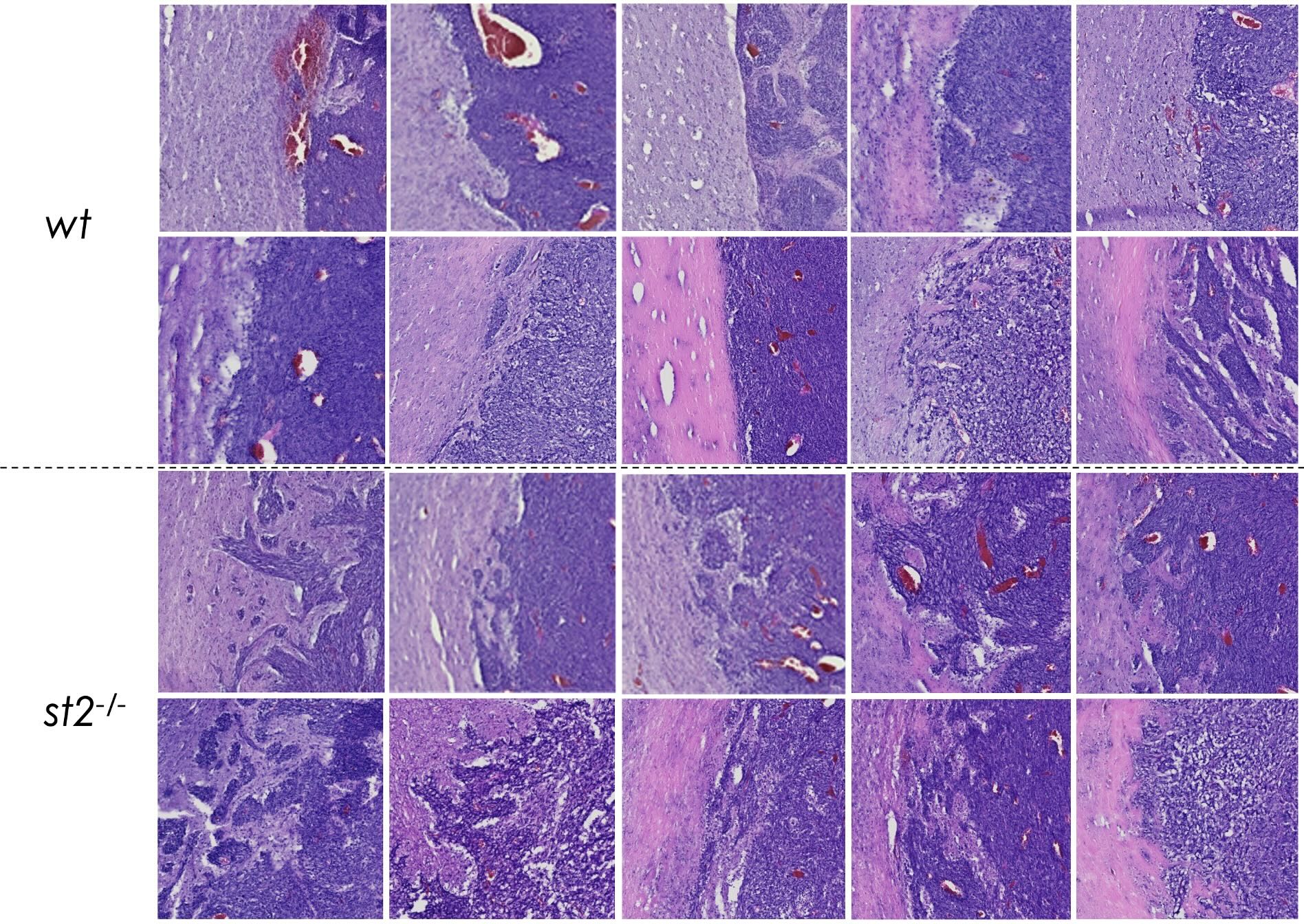
**

**Comparison of the CT-2A-derived tumor and host brain interface in *st2^-/-^* mice and *wt* mice**.

H/E staining of sections through mouse brains with tumors after injecting C2-2A cells. Morphology of tumor-host brain borders, showing more pronounced irregularities in *st2^-/-^* brains (n=10) compared to *wt* brains (n=10). Representative data from three repeats.

**Figure S9**

**
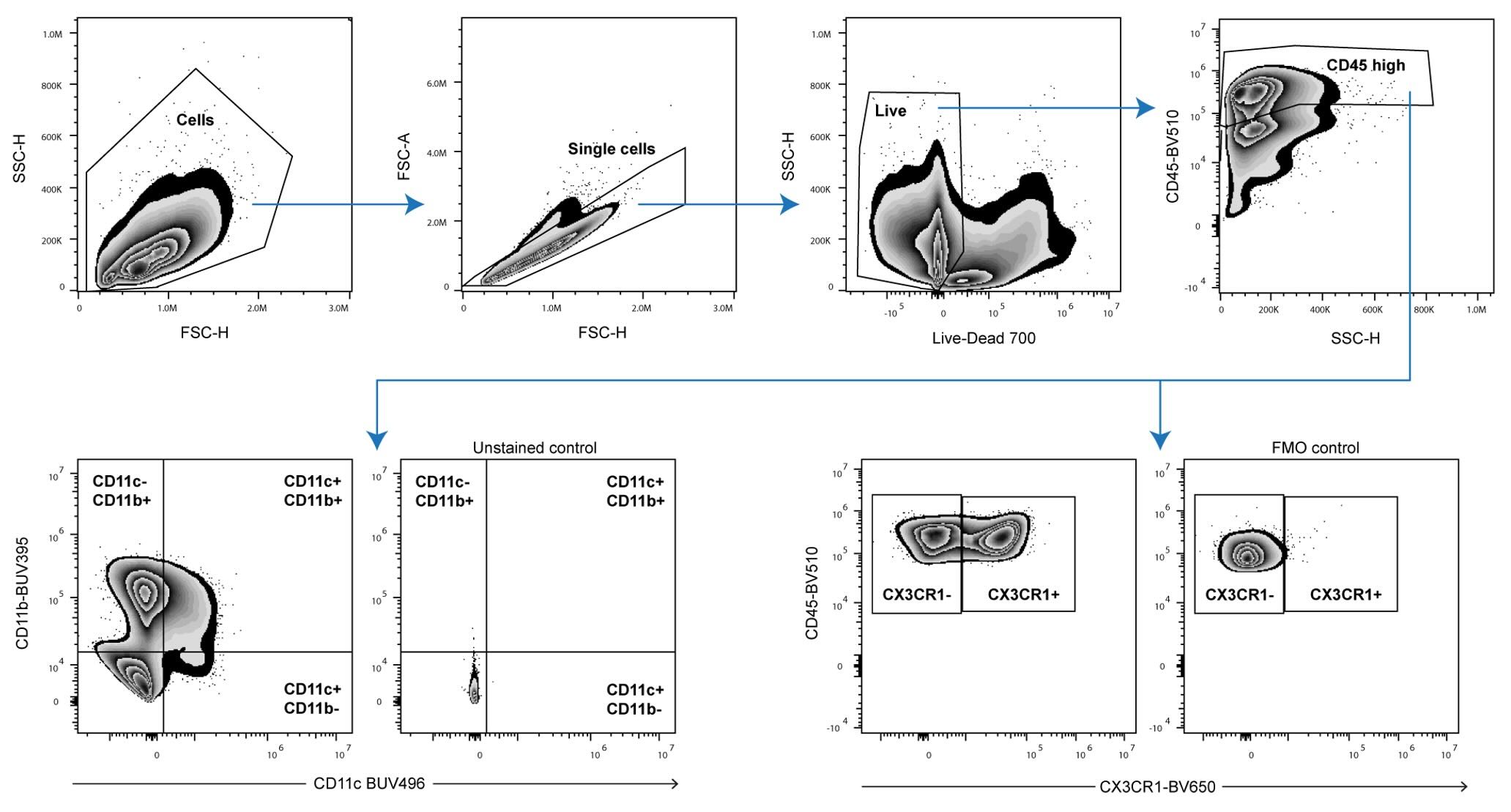
**

**Gating strategy for FACS analysis used to obtain data for myeloid cells.**

The figure illustrates the sequential steps and criteria used to identify and isolate myeloid cell populations from the sample, including the initial scatter plot gating, singlet discrimination, and specific marker-based gating.

**Figure S10**

**
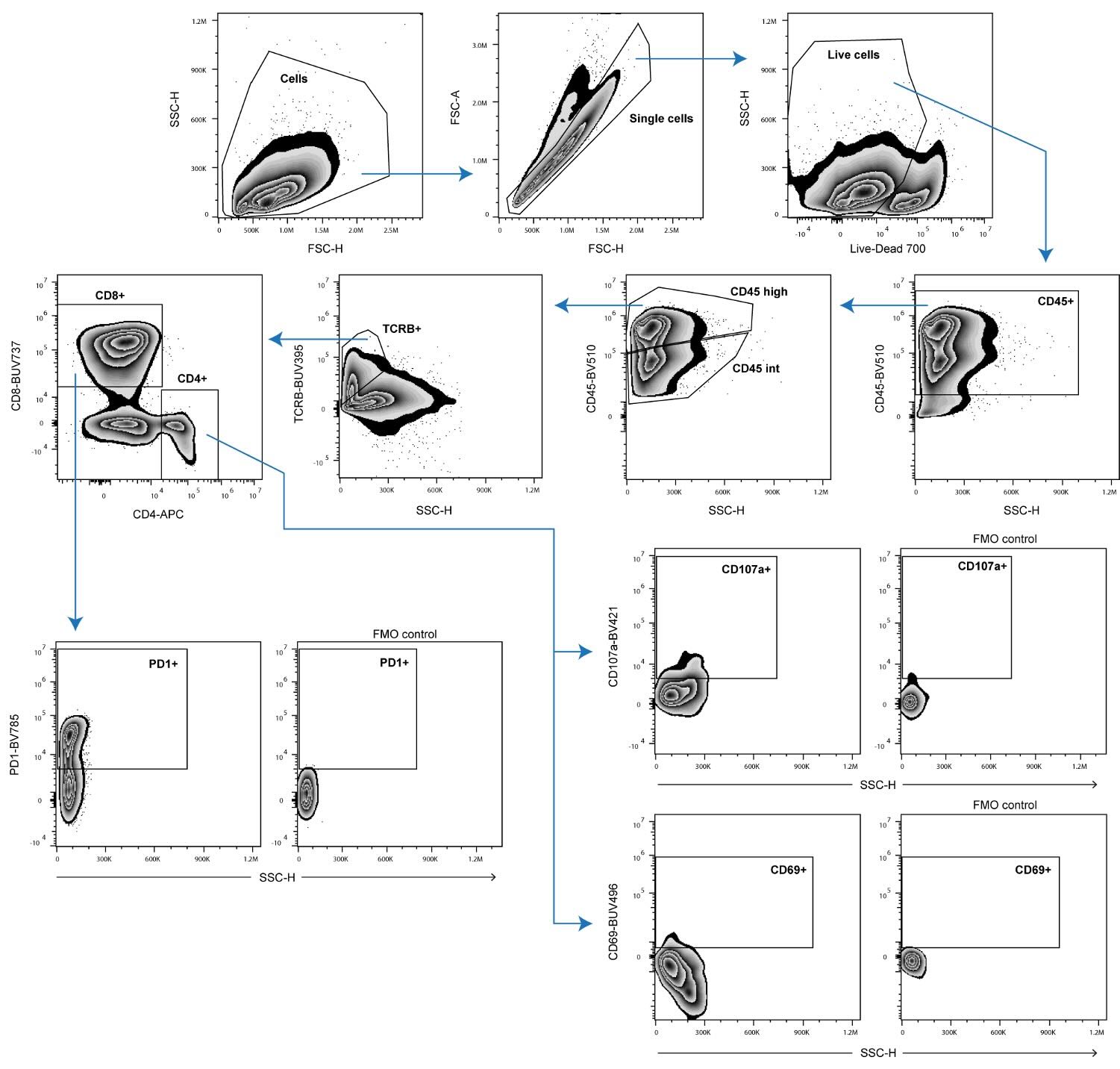
**

**Gating strategy for FACS analysis used to obtain data for T cells.**

The figure illustrates the sequential steps and criteria used to identify and isolate T cell populations from the sample, including the initial scatter plot gating, singlet discrimination, and specific marker-based gating.

**Figure S11**


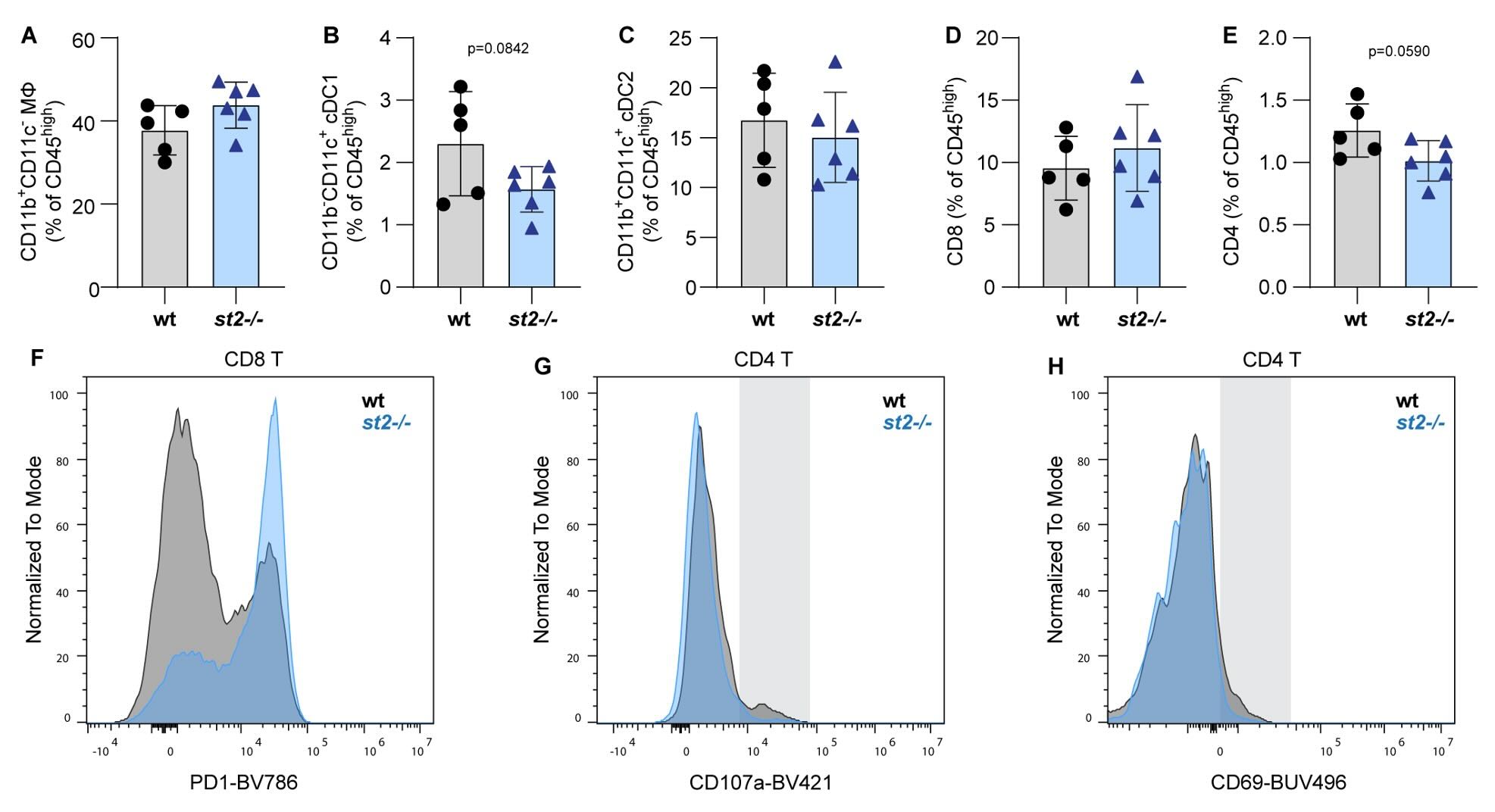


**Percentage of investigated immune populations and histogram representations of immune cell types**

A-E) Analysis of the percentage of investigated immune populations, including macrophages, cDC1, cDC2, CD8 T cells, and CD4 T cells.

The figure also includes histogram representations of PD1 expression on CD8 T cells (F), as well as CD107a (G) and CD69 (H) expression on CD4 T cells. The grey boxes in panels G and H highlight a peak present in wild-type (wt) but absent in ST2-deficient (st2-/-) samples.

**Figure S12**

**
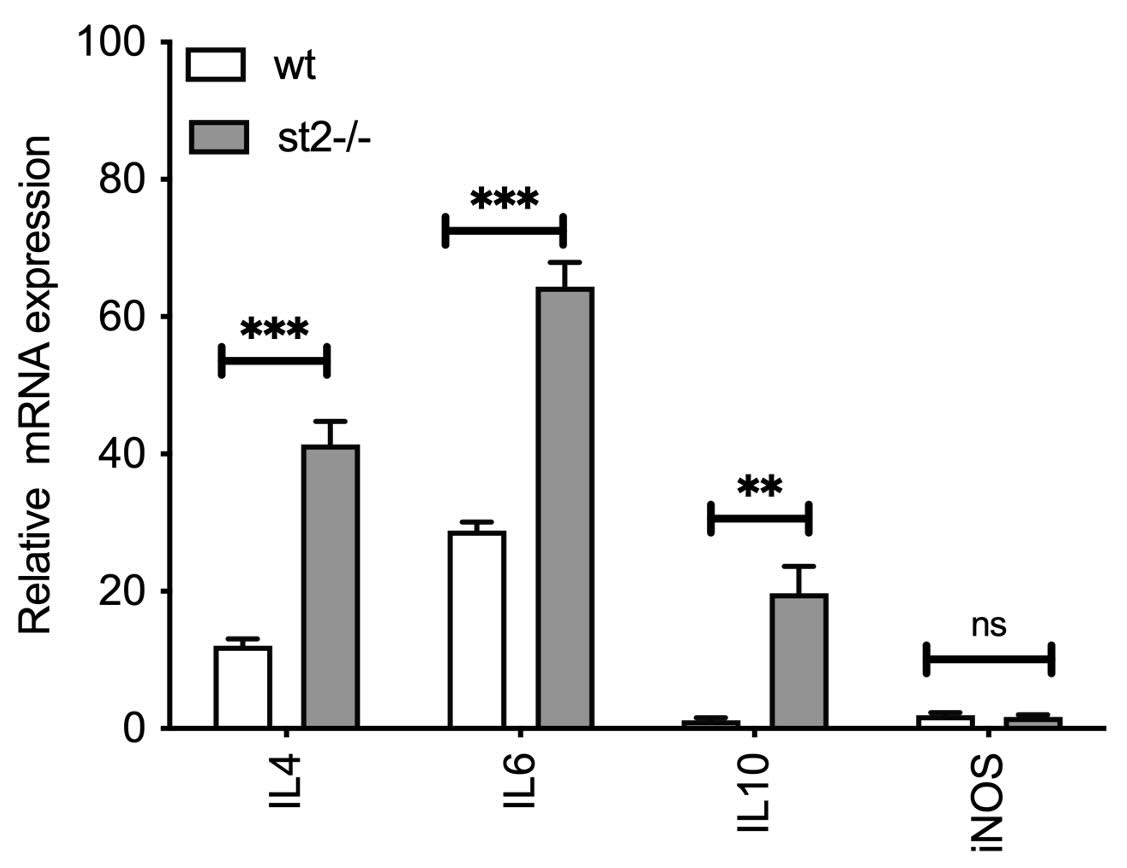
**

**Relative mRNA expression of cytokines in brains of *st2^-/-^* and *wt* mice with CT-2A tumors**

qPCR analysis of cytokines reveals higher mRNA expression of IL-4, IL-6 and IL-10, and unaltered iNOS in tumor tissue from *st2^-/-^* mice compared to *wt* mice in CT-2A syngeneic brain tumors. Data are based on five mice per group and the experiment was repeated twice. Data are presented as mean± SD.

**Supplementary Table S1.**

Primary antibodies

| Antigen | Clone | Company - Catalog number |
| --- | --- | --- |
| Arginase I | A1exF5 | ebioscience- 25-3697-82 |
| CD103 | M290 | BD Biosciences- 564320 |
| CD107a | 1D4B | BD Biosciences- 564347 |
| Cd11b | M1/70 | BD Biosciences- 563553 |
| CD11c | N418 | BD Biosciences- 750450 |
| CD127 | A7R34 | BioLegend- 135016 |
| CD25 | 3C7 | BD Biosciences- 553075 |
| CD4 | RM4-5 | BioLegend - 100533 |
| CD4 | GK1.5 | BD Biosciences- 612952 |
| CD44 | IM7 | BD Biosciences- 564587 |
| CD45 | 30-F11 | BD Biosciences- 563891 |
| CD62L | MEL-14 | BD Biosciences-560516 |
| CD69 | H1.2F3 | BD Biosciences-553235 |
| CD8 | 53-6.7 | BD Biosciences- 612759 |
| CD86 | GL1 | BD Biosciences- 741737 |
| CNPase |  | Sigma-Aldrich - C5922 |
| CX3CR1 | SA011F11 | BioLegend-149033 |
| F4/80 | BM8 | BioLegend- 123111 |
| GFAP |  | Dako - Z033429-2 |
| IA-IE | M5/114.15.2 | BD Biosciences- 746197 |
| IFNg | XMG1.2 | BioLegend- 505808 |
| IgG |  | Invitrogen - A31570 |
| IL-10 | JES5-16E3 | BioLegend- 505033 |
| IL-12 (p40/p70) | C15.6 | BD Biosciences- 554480 |
| IL-17a | TC11-18H10.1 | BioLegend- 506921 |
| IL-33 |  | R&D Systems - AF3623 |
| IL-33 | Nessy-1 | Enzo Life Science–ALX-804-840 |
| IL10 | JES5-16E3 | BioLegend- 505033 |
| iNOS | CXNFT | eBioscience- 53-5920-82 |
| Ki67 | 16A8 | BioLegend- 652423 |
| KLRG1 | 2F1 | BD Biosciences- 564014 |
| LAG-3 | C9B7W | BioLegend- 125223 |
| Ly-6C | HK1.4 | BioLegend- 128041 |
| Ly6G | 1A8 | BD Biosciences- 563005 |
| ST2 |  | Sigma-Aldrich- PRS3363 |
| STEM121 |  | Takara-Bio- Y40410 |
| TCRb | H57-797 | BD Biosciences- 742485 |

**Supplementary Table 2.**

Secondary antibodies.

| Host – reactivity | Fluorochrom | Company - Catalog number |
| --- | --- | --- |
| goat anti-mouse | FITC 488 | Thermo Fisher - 115-095-075 |
| donkey anti-rabbit | FITC 488 | Jackson ImmunoResearch - 711-096-152 |
| donkey anti-goat | Alexa 488 | Thermo Fisher - A-21432 |
| donkey anti-mouse | Alexa 647 | Thermo Scientific - A-31570 |
| donkey anti-rabbit | Alexa 647 | Jackson ImmunoResearch - 711-605-152 |
| donkey anti-goat | Cy3 | Jackson ImmunoResearch - 705-165-003 |
